# Trends in Occupational Mobility of Health Care Support Workers

**DOI:** 10.1001/jamahealthforum.2026.0285

**Published:** 2026-04-10

**Authors:** Jin Jun, Hyeyeon Shin, Alai Tan, Kezia Scales

**Affiliations:** 1 Center for Healthy Aging, Self-Management and Complex Care, The Ohio State University College of Nursing, Columbus; 2 Office of Research, The Ohio State University College of Nursing, Columbus; 3 PHI, New York, New York

## Abstract

This cohort study examines trends in occupational mobility of health care support workers who entered the workforce in young adulthood in the US.

## Introduction

Upward occupational mobility can be a pathway out of poverty, even across generations, by improving income, resources, prestige, and social networks.^1^ In health care, support roles, such as nursing assistants, provide essential care but earn low wages. Although these jobs can be an entry point to higher-paying positions, upward mobility of these workers remains limited.^2,3,4^ Yet empirical evidence on occupational trajectories of health care support workers (HCSWs) over the life course is scarce.^4,5^ This study illustrates occupational changes every 4 years from 1982 to 2022 among individuals who began their careers as HCSWs during young adulthood, a period when most people enter the workforce.^2^

## Methods

This secondary data analysis used the National Longitudinal Survey of Youth 1979 (NLSY79), which followed a cohort of US residents from 1979 to 2022. We selected participants whose job codes matched HCSW roles in 1982 (eg, nursing assistants/aides) when the cohort was between ages 17 and 24 years. We categorized occupational mobility (eMethods and eTable in Supplement 1) as (1) remaining in HCSW roles, (2) lateral moves to other low-wage roles (eg, cashier), (3) upward mobility to health care professionals (eg, nurses), and (4) upward mobility to other higher-wage roles (eg, managers). Covariates included gender, race and ethnicity, citizenship, education, urbanicity, occupational aspirations, parents’ education and occupation, and household income. Ethnicity data were collected from NLSY79 because HCSWs are more likely to be individuals from racial and ethnic minority groups.

Statistical analysis was conducted from May to October 2025. Descriptive statistics (frequencies, proportions, means, and SDs) were used, and an alluvial graph was created to illustrate occupational transitions. All analyses applied sample weights. We adhered to the STROBE reporting guidelines and received exempt status from The Ohio State University Institutional Review Board due to the use of a publicly available dataset. All *P* values were 2-sided, with significance set at *P* = .05. Statistical analyses were conducted using Stata 18 (StataCorp).

## Results

In a study of 7427 individuals, compared with those entering other jobs, HCSWs in 1982 were more likely to be female (82.2% vs 49.1%; *P* < .001), have some college education (39.0% vs 22.6%; *P* < .001), live in urban areas (85.9% vs 79.0%; *P* < .03), aspire to marry and have a family by age 35 years (24.5% vs 13.9%; *P* = .002), and want to be in health care careers (34.5% vs 4.8%; *P* < .001) (Table). HCSWs exhibited substantial occupational changes over the course of 4 decades (Figure). Among those who remained in the workforce, the proportion of those who stayed in HCSW roles declined to approximately 18% by 1990 (ages 25-32 years). Approximately 11% remained in HCSW roles and approximately 43% had transitioned to other low-wage jobs by 2022 (ages 57-64 years). The share of HCSWs who moved to high-wage positions, within and outside of health care, grew steadily up to approximately 37% until 1998, then plateaued.

**Table.** Baseline Participant Characteristics of Health Care Support Workers (HCSWs) Compared With Those Who Entered Other Jobs in 1982^a^

Characteristic	Participants, % (N = 7427)		*P* value
	HCSWs	Other jobs	
Weighted No. of participants	789 588	25 495 176	
Age in 1982, mean (SE), y	20.88 (0.17)	20.70 (0.03)	.28
Race and ethnicity^b^			
Non-Black or non-Hispanic	79.84	78.88	.69
Black or Hispanic	20.19	21.12	
Sex			
Female	82.24	49.11	<.001
Male	17.76	50.89	
Born in US			
Yes	96.81	95.47	.25
No	3.19	4.53	
Highest education			
High school or less	58.21	70.71	<.001
Some college	39.02	22.59	
Bachelor’s degree or higher	2.77	6.70	
Marital status			
Married	23.13	22.58	.87
Not married	76.87	77.42	
Family in poverty status	11.35	15.52	.13
Urban	85.88	14.12	.03
Rural	79.03	20.97	
Father’s highest education			
High school or less	66.13	63.59	.23
Some college	12.78	10.11	
Bachelor’s degree or higher	21.09	26.29	
Mother’s highest education			
High school or less	76.76	74.16	.62
Some college	11.09	10.88	
Bachelor’s degree or higher	12.15	14.96	
Father’s job			
Low-wage other job	69.64	64.64	.23
High-wage other job	30.36	35.36	
Mother’s job			
Low-wage other job	79.39	81.64	.56
High-wage other job	20.61	18.36	
Work they wanted to do at age 35 y (occupational aspiration)			
What they were doing now	6.81	7.45	.002
Some other job	63.21	70.29	
Married and have a family	24.45	13.97	
Other	5.53	8.29	
What job they wanted to do at age 35 y			
HCSW	13.78	2.14	<.001
Health care professional	34.47	4.83	
Low-wage other job	11.31	32.41	
High-wage other job	40.45	59.67	
Military	0	0.95	

^a^ Analyses used National Longitudinal Survey of Youth 1979 round-specific weights to produce population-representative estimates.

^b^ Race and ethnicity categories are represented per the National Longitudinal Survey of Youth 1979.

**Figure.**
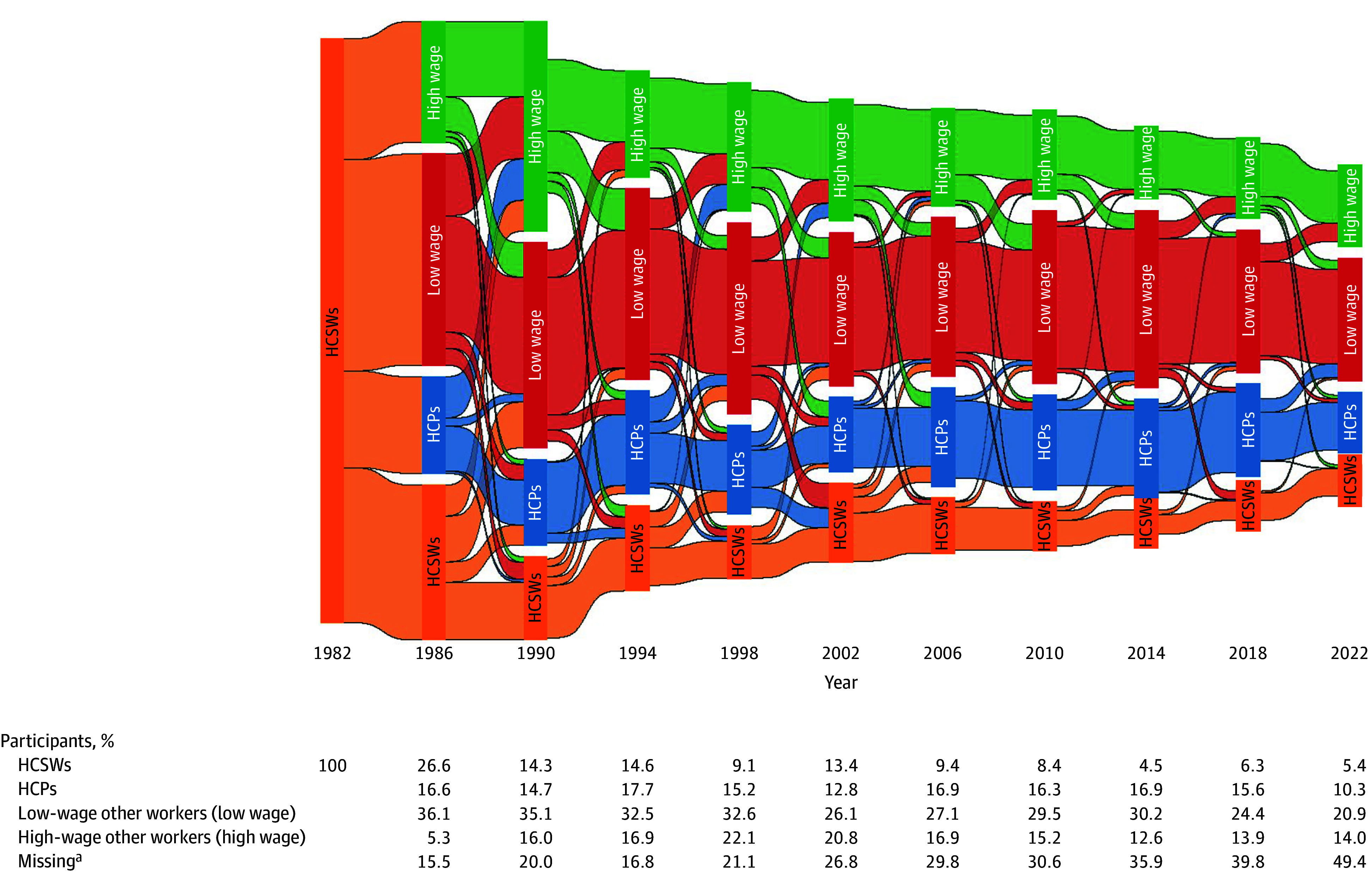
Alluvial Graph of Job Change Trends Among Individuals Who Began Their Careers as Health Care Support Workers (HCSWs) in 1982 ^a^Missing data were excluded to enhance visual clarity. HCPs indicates health care professionals.

## Discussion

Over a 40-year span, the occupational trajectories of HCSWs showed that nearly half of those who began in HCSW roles remained in low-wage positions, while approximately 32% moved upward to either health care professionals or other higher-wage work, reflecting patterns observed in other studies.^2,3,4^ Notably, those remaining in low-wage positions moved out of health care, underscoring the need to examine factors that push them out of HCSW roles and pull them into other roles. Reducing attrition to other low-wage sectors requires health care job improvements, such as higher wages and access to training and professional development opportunities, for those who choose HCSW roles and wish to stay.^5^ Pathways to higher-wage roles are also important for those seeking advancement.^6^

The limitations of the study include a substantial portion of data missing due to nonresponse from participants lost to follow-up, a common issue in longitudinal studies, making it difficult to determine whether employment gaps reflect workforce exit or nonresponse. Although NLSY79 is nationally representative, data were not stratified by occupation, limiting the sample size for HCSWs. Lastly, because this cohort began in 1979, it may not fully capture the dynamic changes in today’s labor market or demographic trends.

## Notes

Supplement 1. eMethods. Occupational Mobility Categories eTable. Occupational codes used to identify Healthcare Support Workers and Healthcare Clinicians eReferences

Supplement 2. Data Sharing Statement
