# Supplementary material for: Trends in Occupational Mobility of Health Care Support Workers
Source: JAMA Health Forum. 2026 Apr 10;7(4):e260285. doi: 10.1001/jamahealthforum.2026.0285 (PMC13069453; doi:10.1001/jamahealthforum.2026.0285)
Supplement: Supplement 1. — eMethods. Occupational Mobility Categories eTable. Occupational codes used to identify Healthcare Support Workers and Healthcare Clinicians eReferences [file jamahealthforum-e260285-s001.pdf]

Supplemental Online Content

Jun J, Shin H, Tan A, Scales K. Trends in occupational mobility of health care support workers. *JAMA Health Forum*. Published online April 10, 2026.
doi:10.1001/jamahealthforum.2026.0285

eMethods. Occupational Mobility Categories
eTable. Occupational codes used to identify Healthcare Support Workers and Healthcare Clinicians
eReferences

This supplemental material has been provided by the authors to give readers additional information about their work.

© 2026 Jun J et al. *JAMA Health Forum*.

eMethods. Occupational Mobility Categories

Participants' current/last occupations were routinely collected as a part of the National Longitudinal Survey of Youth 1979 (NLSY79).[1] Self-reported occupations were coded using the Census classification which was updated periodically from 1982 to 2022. Although the occupation codes evolved to reflect a more complex labor market, the broader occupational categories remained consistent over time, allowing for longitudinal comparisons.[1]

In this study, we used the 1970 Occupational Codes and its categorization[2] from the U.S. Bureau of the Census to initially identify healthcare support workers and other occupational groups as shown in eTable 1. Occupational mobility categories (HSWs, Other Low Wage, Healthcare Professionals, vs. Other Higher Wage) were determined by the authors based on the two-digit Duncan Socioeconomic Index scores[3] associated with the 1970 Occupational Codes.

eTable. Occupational codes used to identify Healthcare Support Workers and Healthcare Clinicians

HSW Roles	Other Low-Wage Roles	Healthcare Professionals	Other Higher-Wage Roles
1970 Census Occupational Codes: The 3-digit 1970 Census classifications (U.S. Census Bureau 1971)[2] are used to code all job and training questions in the 1979-2000 surveys.			
• Dental Assistant (921)<br>• Health Aides (exclude nursing, 922)<br>• Health Trainee (923)<br>• Lay Midwives (924)<br>• Nursing Aides (925)<br>• Practical Nurse (926)	• Clerical and Unskilled Workers (301-395)<br>• Craftsmen and Kindred Workers (401-580)<br>• Operatives, except Transport (601-695)<br>• Transport Equipment Operatives (701-715)<br>• Laborers, except Farm (740-785)<br>• Farmers and Farm Managers (801-802)<br>• Farm Laborers and Farm Foremen (821-824)<br>• Service Workers, except Private Household (901-965)<br>• Private Household Workers (980-984)	• Healthcare Health Diagnosing and Treating Practitioner (061-076)<br>• Health technologists and technicians (080-085)	• Professional, Technical, and Kindred Workers (001-195)<br>• Managers and Administrators, except Farm (201-245)<br>• Sales Workers (260-280)
1980 Census Occupational Code: Beginning with the 1982 survey, the 3-digit 1980 Census codes (U.S. Census Bureau 1981)[4] have been used, in addition to the 1970 codes, to classify occupations of respondents' current or most recent job (also used through the 2000 survey).			
• Dental Assistant (445)<br>• Health Aides (exclude nursing, 446)<br>• Nursing Aides, Orderlies, and attendants (447)<br>• Practical Nurse (366)	• Personal Care and Service Workers (403-407, 456-469)<br>• Protective Service Occupations (413-427)<br>• Food Preparation and Service (433-444)	• Health diagnosing occupations (084-089)<br>• Health assessment and treating occupations (095-097)<br>• Therapists (098-106)	• Executive, Administrative and Managerial Occupations (003-199) except health occupations<br>• Technicians and Related Support

© 2026 Jun J et al. *JAMA Health Forum*.

:	• Cleaning and Building Service Occupations (448-455)<br>• Farming, Forestry, and Fishing Occupations (473-499)<br>• Precision production, Craft, and Repair Occupations (503-699)<br>• Operators, Fabricators, and Laborers (703-999)	• Health technologists and technicians (203-208)	Occupations (213-389)
2000 Census Occupational Code: Surveys starting in 2002, the 2000 Census codes (U.S. Census Bureau 2000)[5] were used to classify occupations of all jobs reported.			
• Nursing, Psychiatric, and Home Health Aides (360)<br>• Occupational Therapist Assistants and Aides (361)<br>• Physical Therapist Assistants and Aides (362)<br>• Massage Therapists (363)<br>• Dental Assistants (364)<br>• Medical Assistants and Other Healthcare Support Occupations (365)	• Protective service (370-395)<br>• Food preparation and serving related (400-416)<br>• Building and grounds cleaning and maintenance(420-425)<br>• Personal care and service (430-465)<br>• Office and administrative support (500-593)<br>• Farming, forestry, and fishing (600-613)<br>• Construction and extraction (620-694)<br>• Installation, repair, and maintenance (700-762)<br>• Production (770-896)<br>• Transportation and material moving (900-975)	• Healthcare practitioners and technical (300-354)	• Management (001-043)<br>• Business and financial operations (050-095)<br>• Computer and Mathematical (100-124)<br>• Architecture and engineering (130-156)<br>• Life, physical, and social services (160-206)<br>• Legal (210-215)<br>• Education, training, and library (220-255)<br>• Art, design, entertainment, and media (260-296)<br>• Sales and related (470-496)

eReferences

1. Bureau of Labor Statistics, U.S. Department of Labor. *National Longitudinal Survey of Youth 1979 cohort, 1979–2022 (Rounds 1–30)*. Center for Human Resource Research, The Ohio State University. Published 2025. Accessed November 4, 2025. https://www.nlsinfo.org/content/cohorts/nlsy79/topical-guide/employment/occupations [nlsinfo.org]
2. Three-digit Industry and Occupation Codes - U.S. Bureau of the Census, *1970 Census of Population Classified Index of Industries and Occupations*, U.S. Government Printing Office, Washington, D.C., 1971.
3. Two-digit Duncan socioeconomic index scores - Albert J. Reiss, Jr., et al., *Occupations and Social Status*, Free Press of Glencoe, New York, N.Y., I961.

© 2026 Jun J et al. *JAMA Health Forum*.

4. United States Bureau of the Census. *1980 Census of Population: Alphabetic Index of Industries and Occupations*. Washington, D.C.: U.S. Government Printing Office, 1981.
5. United States Bureau of the Census. 2000 Census of Population and Housing: Alphabetical Index of Industries and Occupations. Washington, DC: U.S. Government Printing Office; 2000. Accessed November 4, 2025. https://www.census.gov/topics/employment/industry-occupation/guidance/indexes.html

© 2026 Jun J et al. *JAMA Health Forum*.
