# Supplementary material for: Trends in Occupational Mobility of Health Care Support Workers
Source: JAMA Health Forum. 2026 Apr 10;7(4):e260285. doi: 10.1001/jamahealthforum.2026.0285 (PMC13069453; doi:10.1001/jamahealthforum.2026.0285)
Supplement: Supplement 2. — Data Sharing Statement [file jamahealthforum-e260285-s002.pdf]

Data Sharing Statement

Jun. Trends in Occupational Mobility of Health Care Support Workers. *JAMA Health Forum*. Published April 10, 2026. doi:10.1001/jamahealthforum.2026.0285

Data
Data available: Yes
Data types: Other (please specify)
Additional Information: The data used in this study is publicly available on the Bureau of Labor Statistics Website (https://www.bls.gov/nls/).
How to access data: We will make the statistical analysis available upon request.
When available: With publication

Supporting Documents
Document types: Statistical/analytic code
How to access documents: We will make the statistical analysis available upon email request.
When available: With publication

Additional Information
Who can access the data: Anyone requesting the data
Types of analyses: For any purpose
Mechanisms of data availability: via emails
